# Evaluation of Immunoprotective Effects of *Fusobacterium necrophorum* Outer Membrane Proteins 43K OMP, Leukotoxin and Hemolysin Multi-Component Recombinant Subunit Vaccine in Mice

**DOI:** 10.3389/fvets.2021.780377

**Published:** 2021-12-06

**Authors:** Jiawei Xiao, Jiancheng Jiang, Xianjing He, Siyao Zhang, Zhihui Wang, Fengfeng Wang, Lina Wang, Donghua Guo

**Affiliations:** Heilongjiang Provincial Key Laboratory of Prevention and Control of Bovine Diseases, College of Animal Science and Veterinary Medicine, Heilongjiang Bayi Agricultural University, Daqing, China

**Keywords:** 43K OMP, hemolysin, leukotoxin, immunoprotective, *Fusobacterium necrophorum*

## Abstract

We evaluated the efficacy of three vaccine formulations containing different combinations of proteins (43K OMP, leukotoxin recombinant protein PL4 and hemolysin recombinant protein H2) and killed whole cell *Fusobacterium necrophorum* in preventing liver abscess. Four subcutaneous vaccines were formulated: vaccine 1 (43K OMP), vaccine 2 (PL4 and H2), vaccine 3 (43K OMP, PL4 and H2), and vaccine 4 (killed whole bacterial cell). 43K OMP, PL4, and H2 proteins were produced by using recombinant protein expression. To evaluate vaccine efficacy, we randomly allocated 50 BALB/c female mice to one of five different treatment groups: PBS control group, vaccine 1, vaccine 2, vaccine 3, and vaccine 4. Mice were vaccinated three times, with 14 days between each immunization. After immunization, the mice were challenged with *F. necrophorum*. The three key findings of this study are as follows: (1) Vaccine 3 has enabled mice to produce higher antibody titer following bacterial challenge, (2) in the liver pathology of mice, the vaccine 3 liver showed the least pathology, and (3) all four vaccines produced high levels of antibodies and cytokines in mice, but the level of vaccine 3 was the highest. Based on our results, it has been demonstrated that a mixture of *F. necrophorum* 43K OMP, PL4, and H2 proteins inoculated with mice can achieve protection against liver abscess in mice. Our research may therefore provide the basis for the development of a vaccine against *F. necrophorum* bovine infections.

## Introduction

*Fusobacterium necrophorum* is a Gram-negative, anaerobic bacterium. *F. necrophorum* can cause necrotic and purulent diseases in cattle, sheep, pigs and humans (1–5). *F. necrophorum* mainly cause liver abscess in beef cattle and footrot in dairy cows. Liver abscess are almost always caused by co-infection with *F. necrophorum* and several bacterial species, with *F. necrophorum* in the rumen being the main pathogen of liver abscess, followed by *Trueperella pyogenes* (6). In recent years, studies have found that *F. necrophorum* is involved in the occurrence of dairy cow mastitis, endometritis, interdigital dermatitis, and esophageal ulcers (7–11). *F. necrophorum* are very harmful to cattle and sheep farming, the most important of which are liver abscess and footrot caused by *F. necrophorum*, which hasten the culling of animals and reduce their economic value. This has led to a deeper interest in vaccine research for *F. necrophorum* and vaccine development has become urgent.

*F. necrophorum* possesses or secretes a number of virulence factors, which include the following: leukotoxin, endotoxic lipopolysaccharide (LPS), hemolysin, hemagglutinin, capsule, adhesins or outer membrane proteins, platelet aggregation factor, dermonecrotic toxin, and several extracellular enzymes (12). The main virulence factors of *F. necrophorum* are leukotoxin, hemolysin and outer membrane proteins (OMPs). Among them, leukotoxin is an extracellular secretory protein, *F. necrophorum*'s most important virulence factor, which can inhibit the phagocytosis of neutrophils and Kupffer cells (13–15). Studies have shown that the amount of leukotoxin secreted by *F. necrophorum* correlates positively with the severity of liver abscess in cattle (16).

Erythrocytes are able to be lysed by *F. necrophorum* and the virulence factor that performs the main function is the secreted protein hemolysin (17–20). Studies have shown that natural *F. necrophorum* hemolysin can be used to immunize rabbits and has an immunoprotective effect (21). The OMPs of Gram-negative bacteria play an important role in bacterial adhesion and infection in the initial stage of bacterial infection (22–25). The functions of OMPs are mainly structural maintenance, material transport, adhesion and induction of protective immunity. Subcutaneous immunization with recombinant proteins (FimH, LKT, PLO) of three key virulence factors of *E. coli, F. necrophorum*, and *Trueperella pyogenes* was found to be effective in preventing endometritis in cattle (26). We discovered an outer membrane protein of *F. necrophorum* in 2013, designated as 43K OMP. The results of cell adhesion experiments showed that the protein adheres to host target cells (27, 28). Recombinant protein PL4 (60 kDa) of *F. necrophorum* leukotoxin alone induces good immune protection in mice against *F. necrophorum* infection as vaccine (18). We expressed a recombinant protein H2 (30 kDa) of *F. necrophorum* hemolysin that reacts with *F. necrophorum* polyclonal antibodies and has good antigenicity in 2015 (21). In the evaluation of vaccine immunity, cellular and humoral immunity cytokines are usually used as indicators. In our experiments, we measured the cellular immunity-related cytokines IL-2, IFN-γ, IL-1β, the humoral immunity-related cytokines IL-4, IL-10, TNF-α in order to evaluate the effectiveness of the recombinant protein 43K OMP+PL4+H2 vaccine from *F. necrophorum* in immunized mice.

Therefore, our aim was to immunize mice with a mixture of purified *F. necrophorum* recombinant protein 43K OMP, leukotoxin PL4, and hemolysin H2. The immunization effect of 43K OMP, PL4, and H2 was determined by assaying cytokines, antibody titers, liver bacterial load, and pathological findings.

## Materials and Methods

### Ethics Statement

In this experiment, female BALB/c mice, approximately 6 weeks old and weighing 20 g each, were purchased from Changchun Yisi Experimental Animal Technology Co., Ltd. All experiments were approved by the Heilongjiang Bayi Agricultural University, which were conducted in accordance with the regulations of the College of Animal Science & Veterinary Medicine. All efforts were made to minimize animal suffering and to reduce the number of animals used.

### Vaccine Preparation

*Fusobacterium necrophorum subsp. necrophorum* A25286 (purchased from ATCC Company, ATCC 25286, Manassas, VA, USA) (Hereafter referred to as A25) was added to the Fastidious Anaerobe Broth (Qingdao Hope Bio-Technology Co., Ltd, China, FAB) medium at a ratio of 1:100 and cultured in an anaerobic incubator for 24–36 h at 37°C, and grown to an absorbance of 0.6–0.7 at OD_600nm_. Neutral formaldehyde was added to the cultivated *F. necrophorum* to a final concentration of 0.2%, and the inactivation continued for 24 h at 37°C in an anaerobic incubator. The killed whole bacterial cell suspension was inoculated in FAB medium at 1:100 and cultured under anaerobic conditions for 24 h to determine whether bacterial growth occurred. If the bacteria did not grow, the inactivation was considered successful.

The recombinant proteins used in our vaccine preparation include: *F. necrophorum* outer membrane protein 43K OMP, leukotoxin PL4, and hemolysin H2. The results of prokaryotic expression and purification were reported in our previous study (18, 21, 28).

### Immunized Mice

Female BALB/c mice weighing approximately 20 g each were randomly divided into five groups: vaccine 1 (43K OMP), vaccine 2 (PL4 and H2), vaccine 3 (43K OMP, PL4 and H2), vaccine 4 (killed whole bacterial cell) and PBS control group (phosphate buffer saline), with ten mice per group. Animals were housed in individual ventilated caging system (IVC) with wood chip bedding. With SPF class housing conditions, all males were housed in 5 animals/cage. The temperature was maintained at 24 ± 1°C and free access to water and rodent food (Beijing Keao Xieli Feed Co., Ltd., China). Each animal was immunized by multi-point dorsal subcutaneous injection with a volume of 0.25 mL and Immunity 25 μg total protein (Table 1). The first immunization was emulsified with Freund's complete adjuvant, and the second and third immunizations were emulsified with Freund's incomplete adjuvant. The interval between each immunization was 2 weeks.

**Table 1:** The proportion of each component vaccine.

**Grouping**	**Final protein concentration**	**Configuration dose (4 mL)**
Vaccine 1	1 mg/ml	0.08 mL 43 kDa OMP+1.92 mL PBS+2 mL Freund's adjuvant
Vaccine 2	Each protein was 0.5 mg/ml	0.22 mL PL4+0.31 mL H2+1.47 mL PBS+2 mL Freund's adjuvant
Vaccine 3	Each protein was 0.33 mg/ml	0.023 mL 43 kDa OMP+0.13 mL PL4+0.2 mL H2+1.647 mL PBS+2 mL Freund's adjuvant
Vaccine 4	-	2 mL killed whole bacterial cell +2 mL Freund's adjuvant
PBS control group	-	2 mL PBS+2 mL Freund's adjuvant

*Purified protein concentration: 43 kDa OMP: 5.26 mg/mL; PL4: 0.945 mg/mL; H2: 0.647 mg/mL*.

### Bacterial Challenge

The *F. necrophorum* A25 strain was cultivated, bacterial cells were inoculated into pre-reduced, anaerobically sterilized brain-heart infusion (Qingdao Hope Bio-Tcehnology Co., Ltd, China, BHI) broth. The bacterial cells were centrifuged at 3,000 × g for 25 min and suspended to 1 × 10^7^ CFU/mL with physiological saline (Short-term buffering in saline would not cause *F. necrophorum* death). The treated *F. necrophorum* was injected intraperitoneally in mice at a dose of 0.25 mL/mouse. After the mice were challenged, the death of the mice was observed and recorded. Blood is collected from mice, which are euthanized on the 7 days after the challenge. Mice were aseptically dissected at a sterilized ultra-clean workbench.

### ELISA Detection of Mouse Antibody Titer

Vaccine 1 (43K OMP), vaccine 2 (PL4 and H2), vaccine 3 (43K OMP, PL4 and H2), and vaccine 4 (Killed whole bacterial cell). Except for vaccine 4, the respective capture antigens for each group of vaccines were the immunized proteins. The capture antigen of vaccine 4 is the *F. necrophorum*. The coating protein (0.05 mol/L carbonate buffer) was used to dilute the antigen protein to 1 μg/mL. Killed whole bacterial cell was diluted with the coating buffer, and the diluted substrate was added to an ELISA plate at 100 μL per well. The plate was sealed with film and incubated overnight at 4°C.

After coating overnight, the remaining liquid in the well was discarded, and 300 μL of washing buffer (0.05% PBS-T) was added to each well. After washing for 5 min by shaking, the washing buffer was discarded; the washing was repeated five times, and the liquid in the well was patted away after the last wash. Then 200 μL of blocking buffer (5% skim milk) was added to each well, and the plate was sealed and placed in a 37°C incubator for 2 h.

After blocking, excess blocking buffer was discarded, and the plate was washed with washing buffer five times, and excess liquid was patted away. Mouse serum was diluted in 2-fold gradient multiples with PBS and added to the enzyme-labeled plate. Then 100 μL of the diluted mouse serum was added to each well. The plate was sealed and incubated at 37°C for 1 h. Excess liquid was discarded. The plate was washed with washing buffer five times and patted dry. Specific secondary antibodies (dilution 1:5 000), coupled with HRP, were added to wells. For total IgG determination, goat anti-mouse IgG (H+L) -HRP antibodies (Labgic Technology Co., Ltd., China) were buffered into PBS. A volume of 100 μL was added to each well, and the plate was incubated for 1 h at 37°C.

After the secondary antibody incubation, the liquid was discarded, the plate was washed five times, and the liquid in the wells was patted away. Then 100 μL of TMB single-component color developing buffer was added to each well, and the plate was incubated at room temperature for 10–15 min in the dark. Finally, 100 μL of stop buffer (2 mol/L H_2_SO_4_) was added. The absorbance was measured with a microplate reader and when OD450nm>1.0, the serum dilution at this point was the antibody titer.

### Mouse Cytokine Detection

Cytokines (IL-4, IL-10, TNF-α) were estimated in mouse serum to study the humoral mediated immune responses and Cytokines (IL-2, IFN-γ, IL-1β) were estimated in mouse serum to study the cell mediated immune responses. Cytokines associated with the promotion of inflammation were detected. To determine whether mixed immunization with *F. necrophorum* leukotoxin PL4, hemolysin H2, and outer membrane protein 43K OMP can induce both cellular and humoral immunity in mice, we assayed the above six cytokines with ELISA kit (Kete Biotechnology Co., China) (Table 2).

**Table 2:** Standard sample dilution concentration.

**Cytokine**	**1**	**2**	**3**	**4**	**5**
IL-1β	5 ng/L	10 ng/L	20 ng/L	40 ng/L	80 ng/L
IL-2	75 ng/L	150 ng/L	300 ng/L	600 ng/L	1,200 ng/L
IL-4	15 pg/mL	30 pg/mL	60 pg/mL	120 pg/mL	240 pg/mL
IL-10	62.5 pg/mL	125 pg/mL	250 pg/mL	500 pg/mL	1,000 pg/mL
TNF-α	50 ng/L	100 ng/L	200 ng/L	400 ng/L	800 ng/L
IFN-γ	50 ng/L	100 ng/L	200 ng/L	400 ng/L	800 ng/L

### Bacterial Loads in Mouse Liver

Mice liver collected aseptically was weighed and placed in a tissue grinder, and 3 mL of physiological saline was added. The tissue was ground into homogenate, which was diluted with normal saline at ratios of 1: 10, 1: 100, 1: 1 000, and 1: 10 000. A 20 μL volume of the diluted tissue homogenate was spread on anaerobic solid medium and incubated for 24 h. The number of single colonies in the plate was recorded, with the number of colonies between 30 and 200, and the bacterial load of the mouse liver was calculated.

### Histopathology of Liver

Collected liver was soaked in 4% formaldehyde solution for later use. Tissue blocks were trimmed to 1 × 1 × 0.5 cm to make paraffin sections and all liver lobes processed for histological study and stained with Hematoxylin-Eosin staining (H&E) stain. After dyeing, the slices were dehydrated, rendered transparent, mounted and observed under a microscope after drying.

### Statistical Analysis

All experiments were repeated at least three times with similar results. Graphs and data analysis were performed using performed using one-way analysis of variance (ANOVA) using GraphPad Prism 9 (GraphPad Software), using Tukey's *post-hoc* test. The data are expressed as the mean ± standard error of the mean (SEM); *p* < 0.05, *p* < 0.01, and *p* < 0.001 were considered statistically significant.

## Results

### Clinical Symptoms

We recorded the deaths of *F. necrophorum* A25 strain infected mice. The mice were immunized and observed to be free of abnormalities. However, after *F. necrophorum* challenge, all groups showed various degrees of clinical symptoms, including less severe symptoms in the vaccine 3 group compared to vaccine 1 and vaccine 2, with only erected fur that returned to normal after 3 days; Vaccine 4 presents with depression and decreased desire to eat and drink, but returns to normal after a few days; In the PBS group, in additional to the above conditions, the abdomens on both sides were sunken inwards, movement was difficult and three mice died.

### Cytokine Level Detection

**Figure 1:**
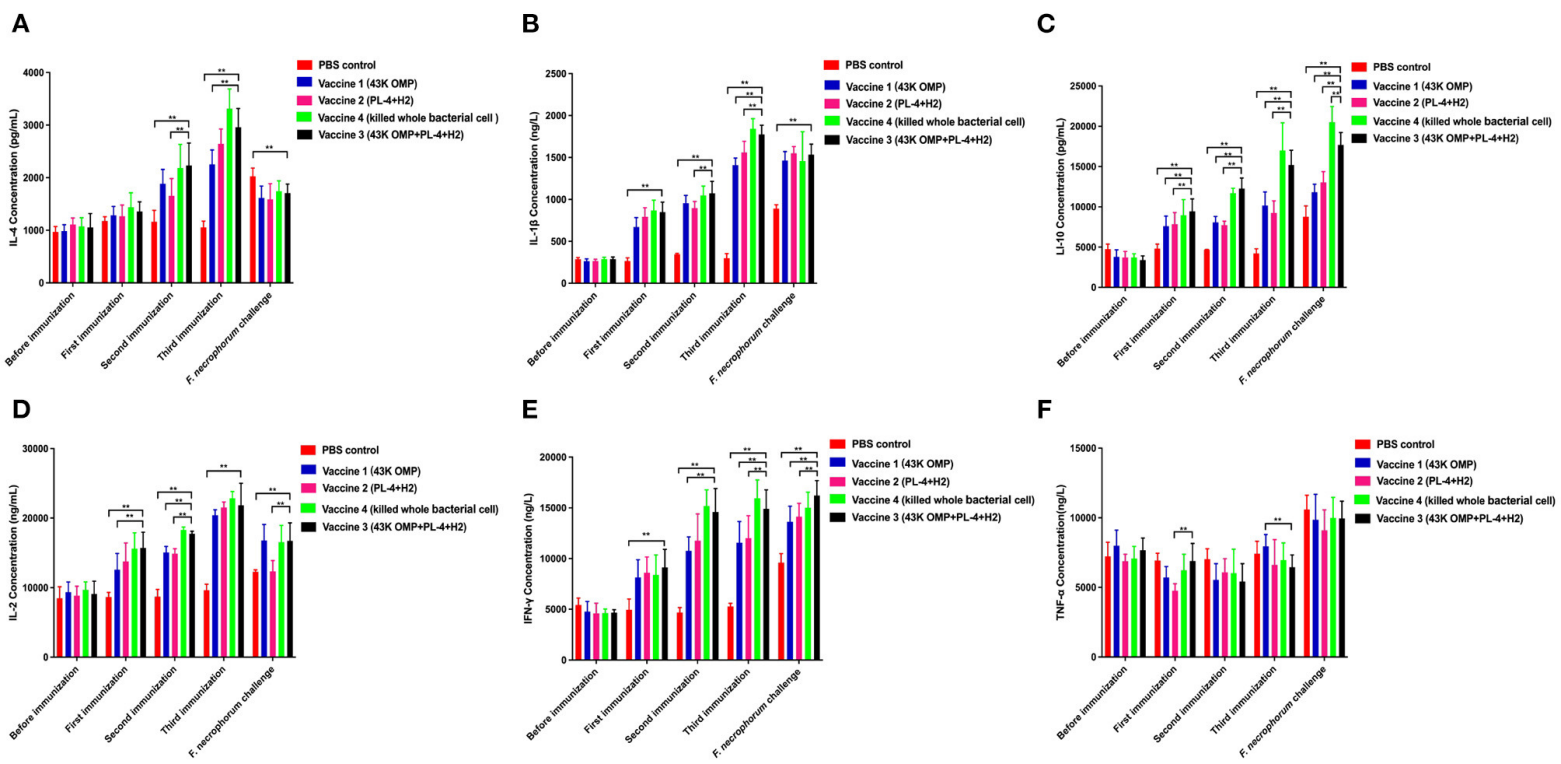
Detection results of cytokines in mouse serum. **(A)** Detection of IL-4 in mice. **(B)** Detection of IL-1β in mice. **(C)** Detection of IL-10 in mice. **(D)** Detection of IL-2 in mice. **(E)** Detection of INF-γ in mice. **(F)** Detection of TNF-α in mice. Data are represented as mean ± SEM of three replicates. *A result significantly different (*P ≤ 0.05 and **P ≤ 0.01) from their respective control. All groups of mice serum were collected the day before the next immunization for cytokine detection.

To determine the pattern of humoral and cellular immune responses following immunization in mice. Cytokines related to cellular (IL-2, IFN-γ, IL-1β) and humoral immunity (IL-4, IL-10) showed an upward trend in the four groups of vaccine immunization (Figure 1). The difference in TNF-α was not particularly significant between several groups while IL-1β showed an increasing trend (Figure 1). After the third immunization, the levels of cytokines in the vaccine 3 were higher than those in the vaccine 1 and the vaccine 2. The difference between the vaccine group and the control group was significant (^**^ *P* ≤ 0.01).

### Antibody Titer Test

**Figure 2:**
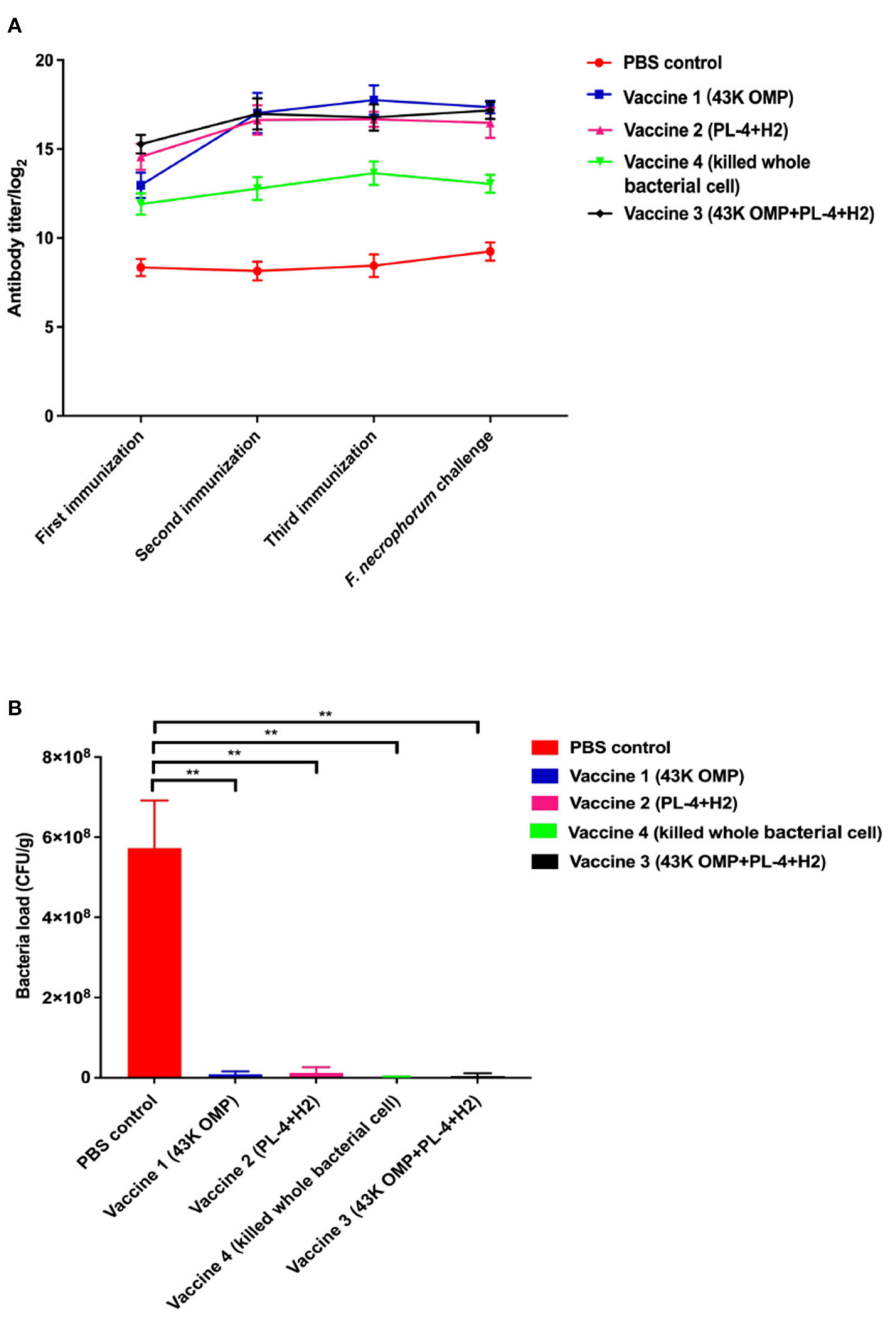
**(A)** Antibody levels in mouse serum. **(B)** Number of bacteria in mouse liver. Data are represented as mean ± S.E.M. of three replicates. *A result significantly different (*P ≤ 0.05 and **P ≤ 0.01) from their respective control.

In past studies for PL4 protein we found that antibody titer reached more than 1:12,800 after 48 days of immunization in mice (18) and the H2 protein has been shown to have excellent antigenicity and can be used in studies of *F. necrophorum* vaccines (21). Based on the above results, the vaccine was immunized in mice BALB/c and the antibody titer was measured after 48 days as follows. After each immunization, mouse serum was collected for antibody titer detection. The results are shown in Figure 2A. The vaccine group produced higher antibody levels against *F. necrophorum*. After 48 days of immunization, Antibody titers reached 1:128 000 in the vaccine 1 (43K OMP), 1:120 000 in the vaccine 2 (PL4 + H2) and 1:120 000 in the vaccine 3 (43K OMP + PL4 + H2) titers in the vaccine 4 (Killed whole cell *F. necrophorum*) were 1:25 600, while antibody titer in the PBS control group was below 1: 400. The 43K OMP protein immunization in mice had a role in immune protection and stimulated the body's immune response. When 43K OMP was combined with PL4 and H2, the immune protection effect was improved.

### Bacterial Load in Mouse Liver

Eight days after infection, the mice were euthanized, and the livers were collected for liver bacterial load experiments. In this experiment, two mice died in the PBS group after the mice were challenged. The results of the mouse liver bacterial load experiments are shown in Figure 2B. The PBS control group had a significantly higher bacterial load of *F. necrophorum* liver compared to the vaccine groups. Of these five groups, vaccine 4 had the lowest liver bacterial load. The bacterial loads among the groups are shown in Table 3.

**Table 3:** Female BALB/c mice liver bacterial load.

**Grouping**	**Bacterial load**
Vaccine 1 (43K OMP)	8 × 10^6^ CFU/g
Vaccine 2 (PL4+H2)	2.34 × 10^6^ CFU/g
Vaccine 3 (43K OMP+PL4+H2)	3.2 × 10^6^ CFU/g
Vaccine 4 (Killed whole bacterial cell)	1.41 × 10^6^ CFU/g
PBS control group	5.91 × 10^8^ CFU/g

### Mouse Liver Histopathology

**Figure 3:**
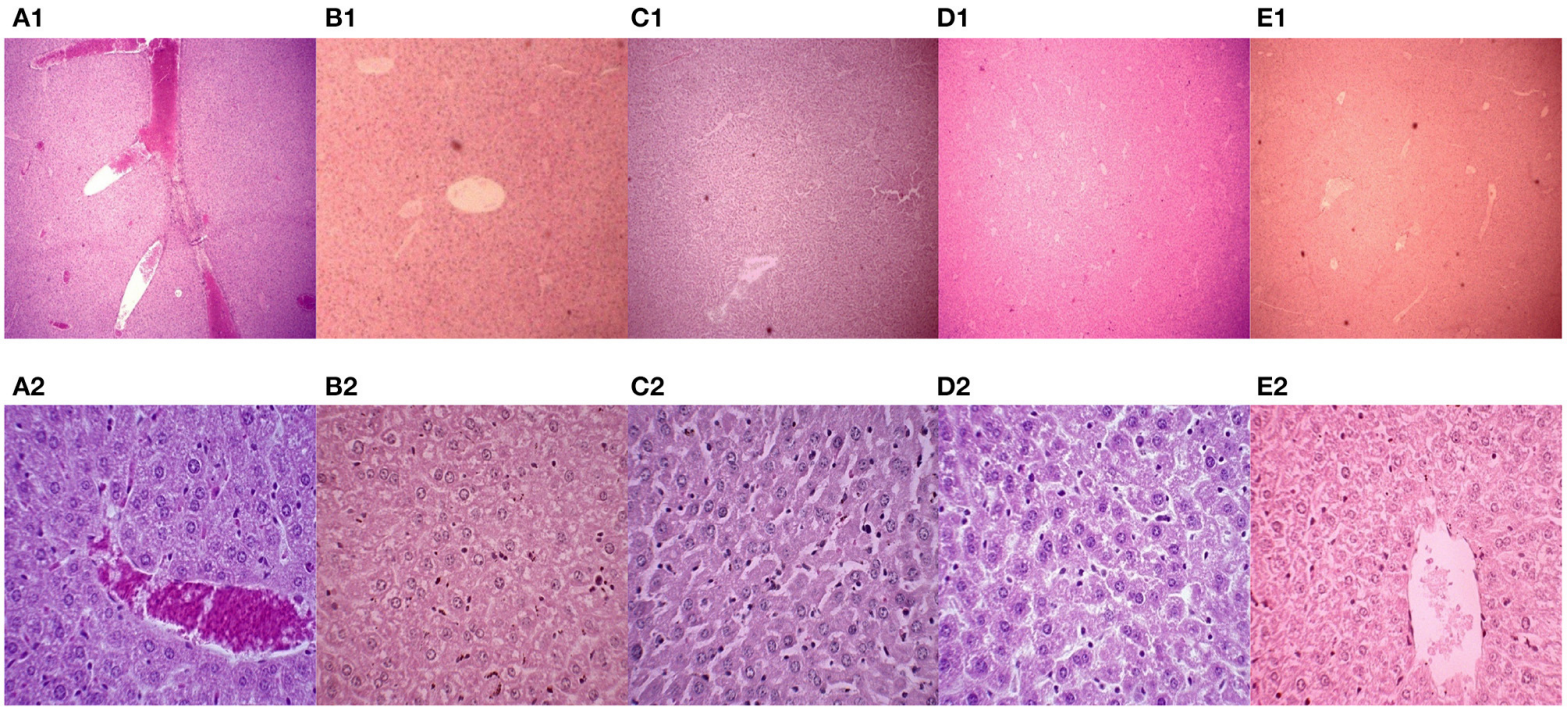
Mouse liver pathology (H.E. 40 ×). **(A1–E1)** Mouse liver pathology (H.E. 40 ×); (A2-E2) Mouse liver pathology (H.E. 400 ×). **(A)** PBS control group. **(B)** vaccine 1 (43K OMP). **(C)** vaccine 2 (PL4 and H2). **(D)** vaccine 4 (killed whole cell *F. necrophorum*). **(E)** vaccine 3 (43K OMP, PL4 and H2).

In this experiment, the mice were euthanized, and the livers were collected. Paraffin sections were made and stained with H&E. The pathological changes in mouse liver were observed under a microscope (Figure 3). The livers in the PBS control group showed severe congestion and bleeding, and clear cellular degeneration. Liver cells in the 43K OMP + PL4 + H2 group, 43K OMP group, and PL4 + H2 group showed slight granular degeneration. Compared with the four vaccines group, the PBS control group had more severe liver lesions.

## Discussion

In this study, we evaluated the effects of four subcutaneous vaccine formulations on liver health in mice based on the results of previous studies on the efficacy of recombinant leukotoxin vaccines from *F. necrophorum* by our class research group. Although the leukotoxin single-component vaccine of Sun et al. (18) had good immune protection, we found that the antibody titers of the multi-component vaccine (43K OMP+PL4+H2 group) were higher than those of the leukotoxin single-component vaccine. This may be due to the fact that *F. necrophorum* can secrete multiple virulence factors, and immune protection is more comprehensive after immunization of mice with recombinant subunit vaccines associated with multiple virulence factors. *F. necrophorum* leukotoxin was shown to prevent metritis with virulence proteins of *Escherichia coli* and *Trueperella pyogenes* as a multi-component vaccine (9, 26, 29). Aeromonas outer membrane protein A (ompA) and hemolysins (hly) were assembled into poly (lactic-co-glycolic) acid (PLGA) carrier vaccines with a strong and stable IgG response (30), which is the protective immune carrier system of Aeromonas. In our study, three recombinant proteins, 43K OMP, PL4, and H2, were purified as vaccine components for immunized mice, and regardless of the 43K OMP group, PL4+H2 group, and 43K OMP+PL4+H2 group, all produced high antibody titers in mice, which were significantly higher than those of killed whole cell *F. necrophorum* group and PBS control group. High levels of a total of IgG help the body resist and clear *F. necrophorum* infections. Such results demonstrated that the multi-component recombinant protein vaccine (43K OMP group, PL4+H2 group, 43K OMP+PL4+H2 group) was able to stimulate the increase of IgG level in mice, and the antibody titers reached the peak after 48 days of immunization.

We measured the levels of the immune-related cytokines IL-4, IL-2, IL-10, IL-1β, IFN-γ, and TNF-α. After the third immunization, IL-4 levels in the 43K OMP + PL4 + H2 group were significantly different compared to the PBS control group (^**^ *P* ≤ 0.01) and showing an upward trend. It has been shown that IL-4 induces differentiation to Th2 (31, 32), gene transcription and anti-apoptosis, and activates B cells to produce more specific antibodies to IgG (33). Elevated IgG levels in the vaccine 4 group were also found in our study. It has been shown that IL-4 induces differentiation to Th2 (30, 31)], gene transcription and anti-apoptosis, and activates B cells to produce more specific antibodies to IgG.

The IL-2 levels in each of the three immunization tended to increase, and the 43K OMP+PL4+H2 group was significantly higher than the PBS control group, which may be due to the fact that the components in 43K OMP+PL4+H2 group stimulate the production of high levels of IL-2 by CD4^+^ and CD8^+^ T cells, which are known to produce high levels of IL-2 under the activation of immune antigens (34–36), in terms of immune stimulation in terms of IL-2, it promotes the proliferation and survival of CD4^+^ and CD8^+^ effector T cells, the differentiation of naive T cells, the growth and differentiation of activated B cells, and the proliferation and function of natural killer (NK) cells (37–41). Thus, mouse T cells may be stimulated by the 43K OMP+PL4+H2 group to produce the corresponding immune response. We suspect that the decrease in IL-4 levels after bacterial challenge, except in PBS control group, is due to the suppression of Th2 cell responses after bacterial infection. IL-10 is a cytokine with anti-inflammatory properties that plays a central role in infections by limiting the immune response to pathogens, thereby preventing damage to the host [42]. In our experiment, the reason for the persistent elevation of IL10 levels after challenge may be that pathogenic bacteria and antigens can activate dendritic cells (DCs), macrophages and neutrophils to trigger IL-10 expression *in vivo* (42–44). IL-4 and IL-10 are produced by Th2 cells and mainly mediate the humoral immune response. In the results of the study, IL-10, INF-γ and TNF-α, all showed an upward trend after challenging bacteria on mice, probably due to bacterial invasion stimulating the expression of organism-related cytokines. IFN-γ in mouse serum was consistently elevated during three immunizations and bacterial challenge. IFN-γ alone cannot fight infection and requires IL-2, IL-4, and GM-CSF to act synergistically (45, 46). Moreover, activated macrophages can release TNF-α, and IL-2 and IFN-γ synergistically can also induce TNF-α secretion (47). IL-1β is mainly produced by monocytes and macrophages, and is overexpressed in chronic local inflammation. Cytokines do not function in isolation in the body; instead, they regulate one another's synthesis and secretion and mutually regulate receptor expression. In this experiment, the levels of cytokines in the vaccine 1, vaccine 2, vaccine 3, and vaccine 4 showed an upward trend after immunization.

After we performed the *F. necrophorum* challenge, Vaccine 3 showed the best immune protection, although no mice died in any group other than the PBS group. This was evidenced by little change in clinical signs and liver pathology in Vaccine 3 and a sustained increase in antibody titers following immunization. In the histopathological findings of the liver, the liver of the PBS control group after challenge showed massive hemorrhage and marked hepatocyte degeneration. The immunized 43K OMP + PL4 + H2 group showed little pathological changes compared to the 43K OMP group, PL4 + H2 group and killed whole bacterial cell group. The PBS control group had the highest liver bacterial load within these groups at 5.91 × 108 CFU/g after 7 days of challenge. The above results demonstrate that 43K OMP + PL4 + H2 group, 43K OMP group, PL4 + H2 group, and killed whole cell *F. necrophorum* group stimulate the immune response in mice. At present, studies on subunit vaccines for *F. necrophorum* are still in the preliminary stage, and since Gram-negative bacteria secrete outer membrane vesicles (OMVs) containing multiple virulence factors, we suspect that significant immune effects may be achieved if the OMVs of *F. necrophorum* can be extracted and prepared as vaccines.

Immunization with 43K OMP + PL4 + H2 group stimulated the immune response in mice better than the 43K OMP group. Because the virulence factors such as leukotoxin, hemolysin, and outer membrane protein were mainly released by *F. necrophorum* infected mice, the recombinant protein of multiple virulence factors of mice was more effective against *F. necrophorum* infection.

The developed multicomponent recombinant subunit vaccine against *F. necrophorum* and outer membrane protein 43 kDa OMP, leukotoxin PL4 and hemolysin H2 can produce higher antibody levels compared to single component subunit vaccines and can induce elevated levels of relevant cytokines in the body to stimulate the body's immune response, with good protection in mice after *F. necrophorum* challenge. This study will provide a good preliminary basis for the development of *F. necrophorum* vaccine.

## Data Availability Statement

The original contributions presented in the study are included in the article/supplementary material, further inquiries can be directed to the corresponding authors.

## Ethics Statement

The animal study was reviewed and approved by Heilongjiang Bayi Agricultural University.

## Author Contributions

DG contributed to research ideas. JX, JJ, and XH contributed to experimental work, manuscript writing data analysis, and revised the manuscript. SZ, ZW, FW, and LW contributed to experimental work. All authors contributed to the article and approved the submitted version.

## Funding

This work was supported by the Natural Science Foundation of Heilongjiang Province, China (Grant No. LH2021C070).

## Conflict of Interest

The authors declare that the research was conducted in the absence of any commercial or financial relationships that could be construed as a potential conflict of interest.

## Publisher's Note

All claims expressed in this article are solely those of the authors and do not necessarily represent those of their affiliated organizations, or those of the publisher, the editors and the reviewers. Any product that may be evaluated in this article, or claim that may be made by its manufacturer, is not guaranteed or endorsed by the publisher.
